# Colecistitis aguda alitiásica por Entamoeba histolytica en lactante

**DOI:** 10.1016/j.aprim.2024.103176

**Published:** 2024-12-16

**Authors:** Luis Ortiz González, Mahmoud Yahdhih Noh, Juan Manuel Contreras Santana

**Affiliations:** a Departamento de Ciencias Biomédicas, Facultad de Medicina y Ciencias de la Salud, Badajoz, España; b Clínica de Pediatría Dr. Contreras, Huelva, España

La colecistitis aguda (CA) alitiásica (CAA) es la forma más frecuente de CA en niños. La mayoría de los casos pediátricos son causados por enfermedades infecciosas, bacterianas, parasitarias o víricas^1^, ^2^, ^3^.

La CA como forma de presentación de una infección por Entamoeba histolytica (EH) es extremadamente rara, especialmente en la población pediátrica. En los niños, la presentación de la CA puede ser inespecífica, con síntomas que incluyen dolor abdominal, fiebre, náuseas y vómitos, que pueden ser fácilmente confundidos con otras causas más comunes de dolor abdominal^4^.

Presentamos el caso de una lactante de 5 meses de edad que presenta un cuadro de 20 días de evolución caracterizado por deposiciones líquidas, verdosas, fétidas y mucosas y, en los últimos 5 días, fiebre alta diaria. En los últimos días, presenta 3-4 episodios diarios de irritabilidad y llanto intenso, de unos 5 min de duración, seguidos de semiología clínica vagal, con palidez, sudoración y somnolencia. En las últimas 24 h tuvo un vómito puntual aislado.

En la exploración física general no presentaba hallazgos patológicos salvo discreto timpanismo abdominal.

Realizamos test de diagnóstico rápido (TDR) inmunocromatográfico para virus digestivos en muestra de heces (rotavirus, adenovirus, astrovirus y norovirus) con resultado positivo para astrovirus. Además, realizamos TDR inmunocromatográfico parasitario (Entamoeba, Giardia y Criptosporidium) con resultado positivo para EH. Después de ello, determinamos los valores de la proteína A de resistencia al Myxovirus (MxA, marcador de infección viral) y de la proteína C reactiva (PCR) conjuntamente en sangre capilar, mediante inmunofluorescencia, con resultados de 234,67 ng/ml (normal < 15 ng/ml) y 32,18 mg/l (normal < 10 mg/l), respectivamente. Un valor elevado de MxA es sugestivo de infección vírica, independientemente de la cuantificación de la PCR^5^. Practicamos una ecografía clínica abdominal donde visualizamos un discreto engrosamiento mural de la vesícula biliar, que no cuantificamos inicialmente; asas de yeyuno algo dilatadas, de contenido anecogénico, con paredes de espesor y peristaltismo normales, y sin imágenes indicativas de invaginación intestinal (fig. 1).

**Figura 1:**
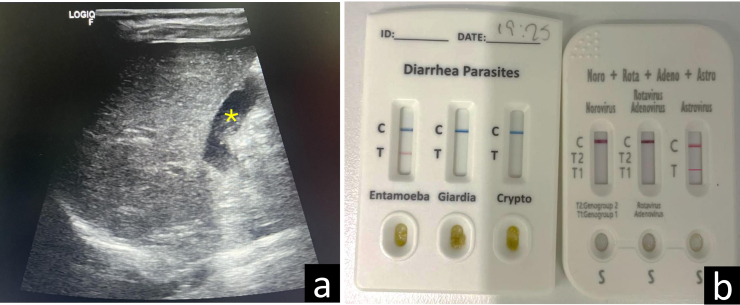
a) Imagen ecográfica obtenida con sonda lineal de alta frecuencia (8-12 MHz), con convexo virtual, mediante corte longitudinal del abdomen a nivel de la línea medio clavicular derecha, donde se identifica la vesícula biliar (*) con discreto engrosamiento mural. b) TDR parasitarios y víricos con positividad para EH y astrovirus.

Consideramos que se trataba un cuadro de coinfección gastrointestinal por EH y astrovirus, por lo que indicamos tratamiento oral con metronidazol y lactasa (la infección por astrovirus induce, más frecuentemente que otros virus, intolerancia secundaria a la lactosa)^5^.

Realizamos control clínico 48 h después, donde el paciente se encuentra afebril, y se pone de manifiesto, de forma evidente en la ecografía, un engrosamiento mural vesicular (4,6 mm) del 50% sobre sobre el límite superior de la normalidad en ayunas (3 mm) (fig. 2), similar al estudio ecográfico previo, sugerente de cuadro de CAA^6^.

**Figura 2:**
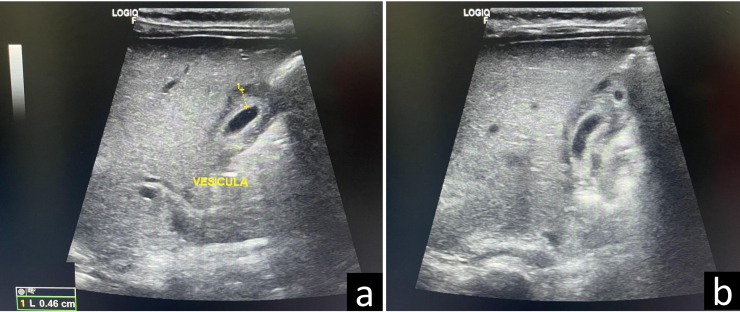
a) Imagen ecográfica obtenida a las 48 h desde el inicio del tratamiento, con sonda lineal de alta frecuencia (8-12 MHz), con convexo virtual, mediante corte longitudinal del abdomen a nivel de la línea medio clavicular derecha, donde se identifica la vesícula biliar, con notable engrosamiento mural (4,6 mm), reflejo de la inflamación activa de la misma. b) Imagen similar a la anterior con un plegamiento de la parte inferior de la vesícula (parte superior de la imagen).

Volvimos a reevaluar al paciente al término del tratamiento (10 días) con normalización de las deposiciones, imagen ecográfica vesicular normal, negativización del TDR de virus digestivos y positividad muy débil del TDR parasitario.

Los episodios de irritabilidad y llanto intenso referidos con anterioridad fueron disminuyendo en frecuencia e intensidad hasta desaparecer antes de la suspensión del tratamiento antibiótico. Estos episodios, la secuencia de la semiología clínica del paciente, su evolución, los TDR y los signos ecográficos, todo ello en el contexto de la atención *point of care* en la Atención Primaria de salud, nos permitieron hacer el diagnóstico clínico de CAA por EH y coinfección por astrovirus.

La elevación específica de la MxA la interpretamos debida a la coinfección por astrovirus.

La identificación precisa de EH como agente causal requiere pruebas serológicas específicas o detección de antígenos en heces mediante TDR. Estos últimos y la ecografía clínica pediátrica son técnicas diagnósticas que, utilizadas de forma conjunta en Atención Primaria, permiten establecer un diagnóstico etiológico, implementar una terapéutica precoz y optimizar los recursos sanitarios.

## Financiación

Los autores manifiestan que no han recibido financiación alguna para la elaboración del manuscrito.

## Consideraciones éticas

Los autores confirman que se han obtenido todos los consentimientos requeridos por la legislación vigente para la publicación de cualquier dato personal o imágenes de pacientes, sujetos de investigación u otras personas que aparecen en los materiales enviados a Elsevier, se han realizado todos los procedimientos éticos y se han respetado los derechos de privacidad de los sujetos humanos.

Los autores conservan una copia escrita de todos los consentimientos y, en caso de que Elsevier lo solicite, aceptan proporcionar las copias o pruebas de que de dichos consentimientos han sido obtenidos.

## Conflicto de intereses

Ninguno.
